# Changes in Patterns of Health Care Use Among Older Adults in Singapore Across a Decade: Repeated Nationwide Cross-Sectional Survey

**DOI:** 10.2196/80157

**Published:** 2026-11-05

**Authors:** Edimansyah Abdin, AshaRani P V, Bernard Tan, Ke Ning, Peizhi Wang, Saleha Shafie, Anitha Jeyagurunathan, Janhavi Ajit Vaingankar, Li Ling Ng, Kelvin Bryan Tan, Paul McCrone, Siow Ann Chong, Mythily Subramaniam

**Affiliations:** 1 Research Division, Institute of Mental Health, 10 Buangkok View, Singapore, 539747, Singapore, 65 63892000; 2 Psychological Medicine, Changi General Hospital, Singapore, Singapore; 3 Health Economist's Office, Ministry of Health, Singapore, Singapore; 4 Institute for Lifecourse Development, Faculty of Education, Health & Human Sciences, University of Greenwich, London, United Kingdom; 5 Saw Swee Hock School of Public Health, National University of Singapore, Singapore, Singapore; 6 Lee Kong Chian School of Medicine, Nanyang Technological University, Singapore, Singapore

**Keywords:** health care use, dementia, aging, network analysis, medical service, health care delivery

## Abstract

**Background:**

There is limited evidence on the patterns of health care use among older adults in Singapore over the past decade.

**Objective:**

This study examines whether the proportion of specific services used within sociodemographic subgroups, the factors associated with health care use, and the network structure of health care use have changed over a decade.

**Methods:**

We used data from 2 repeated nationwide cross-sectional surveys of Singapore residents aged 60 years and older, conducted in 2013 (n=2565) and 2023 (n=2010). Health care service use was obtained from an adapted version of the Client Service Receipt Inventory and analyzed using multivariable logistic regression and network analysis.

**Results:**

The most significant changes between 2013 and 2023 were in the proportions of older adults who had consulted private hospital/clinic doctors (decreased by 4.6 percentage points; n=655, 26.5% vs n=425, 21.9%; *P*=.02), consulted public and private dentists (increased by 6.4 percentage points; n=215, 11.6% vs n=269, 18%; *P*<.001), and had health insurance (increased by 26.9 percentage points; n=1312, 59.9% vs n=1697, 86.8%; *P*<.001). Those diagnosed with high blood pressure and diabetes were more likely to consult primary care doctors, while those diagnosed with dementia and heart problems were more likely to consult public hospital doctors and health workers, visit emergency departments, and require hospitalization services in both 2013 and 2023. We also found that those diagnosed with dementia were more likely to visit intermediate and long-term care services in both 2013 and 2023. Network analysis revealed that there were strong connections between respite care, nursing homes, and hospital admissions in 2023.

**Conclusions:**

This study provides important data on population-level changes in health care usage among older adults across the decade from 2013 to 2023, which will help policymakers ensure better health care delivery for older adults.

## Introduction

### Background

The number of older adults aged 65 years and older is projected to more than double from 771 million in 2022 to 1.6 billion by 2050 [1], suggesting that the world’s population is aging. Aging has been associated with cognitive problems, such as mild cognitive impairment (MCI) and dementia [2,3]. A previous systematic review and meta-analysis found that the global prevalence of MCI was 15.56% [3], while the Global Burden of Disease study suggests that the number of people living with dementia will increase from 57.4 million in 2019 to 152.8 million by 2050 [4]. It has also been reported that aging is associated with an increase in multimorbidity, which requires complex medical management [5]. The complexity of managing multiple health issues and the need for frequent medical attention and management have led to significant increases in health care use, including ambulatory services [6].

Health care use patterns among aging populations are often interpreted through the Andersen Behavioral Model of Health Services Use, which emphasizes the interplay of predisposing characteristics (such as age, gender, ethnicity, and education level), enabling factors (including health insurance coverage, income, and geographical access to care), and need factors (eg, the presence of chronic conditions, such as dementia, hypertension, and diabetes) [7,8]. Although this framework has guided international research for decades, population-level evidence on how these factors evolve over time in multiethnic Asian contexts remains limited. Singapore provides a unique opportunity to examine how predisposing, enabling, and need factors shape health care use patterns across a decade of rapid demographic change and substantive policy reform.

Singapore is a city-state in Southeast Asia inhabited by a multiethnic population that is predominantly Chinese (74%), followed by Malay (13.5%), Indian (9%), and other ethnic groups (3.4%). Singapore is among the fastest-aging countries in the world. The country is experiencing rapid population aging, with the proportion of citizens aged 65 years and above projected to increase from 12.4% in 2014 to 24.1% by 2030 [9,10]. To meet the needs of an aging population, the government has formed a Ministerial Committee on Aging (MCA) to coordinate government policies and programs relating to population aging in Singapore. The MCA has launched 2 Action Plans for Successful Aging in 2015 and 2023 as part of the national blueprint to enable Singaporeans to age well. These include increasing access to aged care services, improving health care affordability through the Pioneer Generation (PG) and Merdeka Generation (MG) packages, providing more senior-friendly homes, and enhancing public infrastructure [9,11].

Singapore’s health care system is a mixed public-private model [12]. Public institutions such as primary care polyclinics and hospitals provide most of the subsidized care through schemes like MediShield Life and CareShield Life, while private clinics and hospitals operate on a fee-for-service basis. To strengthen preventive care, the Ministry of Health launched HealthierSG, a national strategy that encourages enrollment with family doctors and closer integration of public and private providers [13]. The intent is to ensure regular screening, referrals to lifestyle interventions, and management of chronic conditions.

PG and MG packages were introduced by the government to support older adults with their medical bills and provide additional benefits, including annual MediSave top-ups, MediShield Life and CareShield Life premium subsidies, and outpatient care subsidies. CareShield Life, introduced in 2020, is the enhanced successor to ElderShield. It offers higher monthly payouts and lifetime coverage for individuals with severe disabilities; however, it applies only to those who turned 30 years old in 2020 onward or later. ElderShield is a long-term care insurance scheme for Singapore citizens and permanent residents who turned 40 years old before 2020, providing monthly cash payouts to support long-term care needs. Older cohorts may remain on ElderShield unless they opt into CareShield Life. MediShield Life and CareShield Life are national health and long-term care insurance schemes, respectively.

Given the demographic changes and numerous government initiatives implemented in recent years, it is important to examine how health care use patterns among older adults have evolved across this decade of demographic and policy change and what sociodemographic and clinical factors explain persistent disparities in service use.

### Objectives

The objective of the study is to examine changes in health care use patterns among older adults between 2013 and 2023. Specifically, we aim to determine whether the proportion of specific services used within sociodemographic subgroups has changed; the factors associated with health care use have shifted; and the network structure of health care use has evolved over a decade.

## Methods

### Study Design and Setting

The Wellbeing of the Singapore Elderly (WiSE) 2023 study was conducted following the same methodology as the WiSE 2013 study [14,15]. WiSE 2023 and WiSE 2013 are population-based, cross-sectional epidemiological studies that included Singapore residents aged 60 years and older living in Singapore. These surveys used a sampling frame based on a national population registry database of all citizens and permanent residents in Singapore, which is updated regularly. A probability sample was selected using a disproportionate stratified sampling design with 12 strata defined by ethnicity (Chinese, Malay, Indian, or others) and age group (60‐74 y, 75‐84 y, 85 y and older). Residents aged 85 years and older, Malays, and Indians were oversampled to ensure sufficient sample size for subgroup analysis.

### Ethical Considerations

The study procedures were approved by the relevant ethics committees (National Healthcare Group [NHG] Domain Specific Review Board [DSRB]; Ref: 2020/01400), and all participants or their legally acceptable representatives (where applicable) gave written informed consent before the initiation of the study. All participant data were deidentified prior to analysis, with no personally identifiable information retained. Data were stored on secure, password-protected servers accessible only to authorized members of the research team. Participants received a cash reimbursement of SGD $50 (SGD $1=US $0.78 as of October 12, 2026) upon completion of the interview.

### Outcomes

Information on health care service use was obtained from an adapted version of the Client Service Receipt Inventory (CSRI) [16]. The CSRI is a standardized instrument used to collect detailed information on the types and frequency of health and social care services used by respondents over the past 3 months. Data on health care use were self-reported by participants. In this study, we focused on direct medical care services, which refer to care provided by health professionals in the public or private sector, in outpatient or inpatient settings. This included health care services from public primary care doctors (polyclinic doctors), public hospital doctors (ie, doctors employed by the government), other public hospital health care workers (eg, physiotherapists, psychologists, nurses, medical social workers), private hospital/clinic doctors (eg, general practitioners [GPs]), other private health care workers (eg, physiotherapists, psychologists, nurses), public and private dentists, traditional healers, hospitalization in public or private hospitals, health insurance, and admissions to the emergency department.

### Covariates

Data on health conditions were captured by asking participants whether a doctor had ever diagnosed them with any of the following conditions: dementia, hypertension (high blood pressure [HBP]), heart problems, stroke, diabetes, or transient ischemic attacks (TIAs). Multimorbidity (label as Morbidity in Figure 1) was defined as the presence of three or more chronic conditions. Data on sex, age group (60-74, 75-84, ≥85), ethnicity (Chinese, Malay, Indian, and others), marital status (never married, married, widowed, divorced/separated), educational level (none, some but did not complete primary, primary, secondary, and tertiary), employment status (employed, unemployed, and economically inactive, ie, homemakers and retirees), income (received income or no income), and smoking status(ever or never smoked) were also collected. Consistent with the Andersen Behavioral Model of Health Services Use, the sociodemographic variables (sex, age, ethnicity, educational level, and employment status) were treated as predisposing factors, and doctor diagnosed chronic conditions (dementia, hypertension, heart problems, stroke, diabetes, and TIAs) as need factors, whereas enabling factors (national health insurance and long-term care insurance coverage and related subsidies) were examined at the population and policy levels; this framework guided the selection of correlates entered into the multivariable models and the interpretation of the observed changes.

### Statistical Analysis

Statistical analyses were carried out using the Stata (version 13.0; StataCorp LLC) and R (version 4.3.1; R Foundation for Statistical Computing). To account for the stratified, disproportionate sampling design and ensure that the survey estimates were representative of the older adult population in Singapore, weighted survey data were used to estimate health care service use and to describe the sociodemographic profile of the older adult population. A *χ*^2^ test was used to examine changes in the proportion of health care use between the 2 surveys and within each sociodemographic subgroup. A series of multivariable logistic regression analyses were used to examine sociodemographic and clinical correlates of each health care service use within each survey year. Standard errors for logistic regression analyses were estimated using a Taylor series linearization method, while *χ*^2^ tests were Rao-Scott corrected to obtain design-adjusted test statistics and *P* values, accounting for the complex survey design. A network analysis was used to explore the network structure of health care service use and the centrality indices by fitting Ising models to the data using the *IsingFit* R-package [17] and subsequently visualized and assessed the accuracy of edge weights by nonparametric bootstrapping using the *qgraph* and *bootnet* R-packages [18] version 4.3.1. All differences were evaluated using 2-sided tests, with statistical significance set at *P*<.05.

## Results

### Descriptive Statistics

A total of 4575 older adults were included in the final sample, with response rates of 65.6% and 62.7% in 2013 and 2023, respectively. Multimedia Appendix 1 shows the sociodemographic distribution of the older adult population in 2013 and 2023. The most frequently reported health care use categories among older adults in 2023 were primary care doctors (n=932, 44.5%), followed by public hospital doctors (n=678, 31.3%) and private hospital/clinic doctors (n=425, 21.9%; Table 1).

**Table 1.** Percentage of health care use among older adults in 2013 and 2023.

Service	2013, n (%)		2023, n (%)		% Difference	*P* value
Primary care doctors	1082 (42.2)		932 (44.5)		2.4	.28
Public hospital doctors	749 (28.6)		678 (31.3)		2.7	.17
Other public hospital health care workers	121 (4.0)		157 (5.9)		1.9	.04
Any public hospital doctors or health care workers	777 (29.4)		698 (32.2)		2.8	.16
Private hospital/clinic doctors	655 (26.5)		425 (21.9)		–4.6	.02
Other private health care workers	29 (1.6)		31 (1.6)		–0.1	.90
Any private hospital/clinic doctors or health care workers	673 (27.6)		438 (22.3)		–5.4	.005
Public and private dentists	215 (11.6)		269 (18.0)		6.4	<.001
Traditional healers	144 (10.4)		105 (8.4)		–2.0	.14
Day care center	19 (0.7)		32 (1.2)		0.5	.22
Respite care and nursing home	24 (0.7)		33 (0.7)		0.0	.94
Intermediate or long-term care	43 (1.4)		65 (1.9)		0.5	.26
Medications	2109 (80.9)		1620 (78.1)		–2.8	.13
Hospitalizations	156 (4.5)		152 (4.4)		–0.1	.95
A&E^a^	135 (4.3)		129 (5.1)		0.7	.40
Health insurance	443 (25.7)		528 (36.8)		11.2	<.001
MediShield/MediShield Life (from November 1, 2015, onward)	1127 (56.6)		1626 (89.7)		33.1	<.001
ElderShield	442 (24.1)		783 (51.6)		27.5	<.001
Any health insurance	1312 (59.9)		1697 (86.8)		26.9	<.001

a A&E: accident and emergency.

### Changes in the Proportion of Health Care Use Among Older Adults Between 2013 and 2023

The percentage of older adults who consulted other public hospital health care workers increased significantly from 4.0% in 2013 to 5.9% in 2023 (increased by 1.9 percentage points; *P*=.04). The most significant changes between 2013 and 2023 were in the proportions of older adults who had consulted private hospital/clinic doctors (decreased by 4.6 percentage points; *P*=.02), who had consulted public or private dentists (increased by 6.4 percentage points; *P*<.001), and who had health insurance (increased by 26.9 percentage points; *P*<.001), including MediShield and ElderShield.

Table 2 shows the changes in the percentage of health care use within each sociodemographic subgroup between 2013 and 2023. The percentage of Indians who had consulted primary care doctors increased significantly from 46.8% in 2013 to 54.2% in 2023 (an increase of 7.4 percentage points; *P*=.02), while the percentage of Malays who had consulted public hospital doctors and health workers increased significantly from 22.2% in 2013 to 28.3% in 2023 (an increase of 6.1 percentage points; *P*=.02). The largest reduction in private hospital/clinic consultations was observed among those who had below primary education, those with secondary education, those who were employed, and those aged 60 to 74 years. The largest increase in consultations with government and private dentists was among homemakers, those with no formal education, those with below primary education, and the widowed. For intermediate and long-term care, the largest increase was observed among those with no formal education, those with below primary education, those aged 75‐84 years old, and Indians.

**Table 2.** Changes in the percentage of health care use within each sociodemographic subgroup between 2013 and 2023.

Characteristics	Primary care doctors			Public hospital doctors and health workers			Private hospital/clinic doctors and health workers			Public and private dentists			Hospital admissions			A&E^a^			Traditional healers			Intermediate and long-term care			Health insurance		
	T1^b^	T2^c^	%^d^	T1	T2	%^d^	T1	T2	%^d^	T1	T2	%^d^	T1	T2	%^d^	T1	T2	%^d^	T1	T2	%^d^	T1	T2	%^d^	T1	T2	%^d^
Age group (y)																											
60-74	40.8	44.1	3.3	27.3	30	2.7	28.7	21.5	–7.2^e^	13.4	19.3	5.9^e^	3.1	3.4	0.3	3.7	3.9	0.2	11.6	8.6	–3	0.8	0.5	–0.3	67.8	88.9	21.1^e^
75-84	47.7	48	0.3	35.1	38.5	3.4	25.1	24.8	–0.3	7.2	14.9	7.7^e^	7.9	6.2	–1.7	5	8.1	3.1	6.7	9.2	2.5	1.5	5.5	4^e^	40.7	80.5	39.8^e^
≥85	41.8	39.1	–2.7	37.5	39.5	2	22.2	24.7	2.5	3.1	11.9	8.8^e^	10.5	11.3	0.8	11	10.7	–0.3	6.5	3.3	–3.2	9.2	8.6	–0.6	19	80.5	61.5^e^
Sex																											
Male	42.5	45.6	3.1	28.3	33	4.7	25.2	20.2	–5	13	17.5	4.5	5.7	5.7	0	4	6.2	2.2	7.7	6.1	–1.6	1.6	1.6	0	66.5	89.5	23^e^
Female	41.9	43.6	1.7	30.2	31.5	1.3	29.6	24.1	–5.5^e^	10.6	18.5	7.9^e^	3.5	3.3	–0.2	4.6	4	–0.6	12.5	10.4	–2.1	1.2	2.2	1	54.6	84.4	29.8^e^
Ethnicity																											
Chinese	42.5	44.2	1.7	29.6	32.1	2.5	27.8	22.7	–5.1^e^	12.4	20	7.6^e^	4.2	3.9	–0.3	4.3	5	0.7	12.1	9.5	–2.6	1.5	2	0.5	60.8	86.5	25.7^e^
Malay	36.8	40.9	4.1	22.2	28.3	6.1^e^	26.1	20	–6.1^e^	5.1	7.1	2	4.4	5.9	1.5	3.6	4.5	0.9	2	3.2	1.2	0.7	1.5	0.8	48.7	85.4	36.7^e^
Indian	46.8	54.2	7.4^e^	34.7	36.5	1.8	25.6	24.8	–0.8	9.6	14.9	5.3^e^	6.5	9.1	2.6	5.7	8.5	2.8	2.5	5.1	2.6^e^	0.7	1.8	1.1^e^	61.3	89.4	28.1^e^
Others	39.2	46.4	7.2	44.1	48.3	4.2	38.1	3.4	–34.7^e^	19.4	2.6	–16.8^e^	8.7	0.01	–8.69^e^	7.5	0	–7.5^e^	—^f^	—	—	—	—	—	72.2	99.3	27.1^e^
Marital status																											
Never married	30.9	42.3	11.4	22.7	28.1	5.4	23.6	23.7	0.1	17.4	14.7	–2.7	3	2.2	–0.8	2	3.9	1.9	7.2	12.2	5	0.1	1.8	1.7	64	87.3	23.3^e^
Married	43.7	44.6	0.9	29.7	32.5	2.8	29.9	23.2	–6.7^e^	13.3	19.2	5.9^e^	4.8	5.1	0.3	4.1	5	0.9	10.8	7.9	–2.9	1	1.5	0.5	64.9	88.4	23.5^e^
Widowed	42.9	44.9	2	30.9	33.7	2.8	24.9	20.9	–4	6.2	16.1	9.9^e^	4.4	3.9	–0.5	5.9	6.7	0.8	10.2	8.1	–2.1	3.2	4.1	0.9	42.7	80.6	37.9^e^
Divorced/separated	37.1	47.2	10.1	30.5	31.1	0.6	19.7	14.5	–5.2	6.9	16.3	9.4	3.3	2.3	–1	5.1	3	–2.1	11.6	9.2	–2.4	0.3	0.2	–0.1	65.6	89	23.4^e^
Education																											
None	44.2	40.4	–3.8	30.1	31.8	1.7	24.7	30.7	6	4	15.5	11.5^e^	5.2	7.8	2.6	7.8	5.6	–2.2	9.8	13.1	3.3	2.2	6.1	3.9^e^	38.8	71.2	32.4^e^
Some, but did not complete primary	44.6	43.2	–1.4	24.8	29.4	4.6	29	20.4	–8.6^e^	7.2	16.1	8.9^e^	2.9	6.1	3.2	3.8	6.3	2.5	9.7	5.5	–4.2	1.4	4.4	3^e^	47.6	81	33.4^e^
Primary	41.6	47.5	5.9	27.5	31.9	4.4	24.8	22.8	–2	6.3	9.8	3.5	4.7	3.7	–1	3.3	4	0.7	10.6	8.1	–2.5	1.6	0.6	–1	56.6	80.5	23.9^e^
Secondary	41.2	47.5	6.3	29.2	31.2	2	29	20.2	–8.8^e^	19.5	20.3	0.8	5.4	3.1	–2.3	4.1	4.4	0.3	13.9	9.8	–4.1	1	1.1	0.1	78.8	94.4	15.6^e^
Tertiary	36.7	39.1	2.4	41.6	38	–3.6	31.5	23.7	–7.8	26.3	30	3.7	4.2	4.2	0	3.2	6.1	2.9	5.8	8.2	2.4	0.2	0.8	0.6	84	97.8	13.8^e^
Employment																											
Employed	37.1	41.6	4.5	21.9	25.4	3.5	30.7	22.4	–8.3^e^	15.9	19.2	3.3	2.2	2.5	0.3	2.8	3.9	1.1	12.3	8.8	–3.5	—	—	—	74.9	91	16.1^e^
Unemployed	64.8	53.8	–11	33.1	36.6	3.5	24.6	9.2	–15.4	15.5	15.6	0.1	0	15.6	15.6	0	13.9	13.9	7.9	12.2	4.3	—	—	—	69.7	86.3	16.6
Homemaker	40.2	45.8	5.6	29.8	28.6	–1.2	32.1	27	–5.1	7.7	20.2	12.5^e^	3.8	5.2	1.4	5.2	2.6	–2.6	11.3	9.2	–2.1	1.2	2.8	1.6	47.1	78.8	31.7^e^
Retired	46.9	47.1	0.2	35.3	41	5.7	21.9	20.7	–1.2	10.7	16.1	5.4^e^	7.1	5.7	–1.4	5.2	7.2	2	8.1	7.6	–0.5	2.7	3.7	1	55.1	86.1	31^e^
Income
Received no income	43.8	34	–9.8	38.1	28.4	–9.7	19.7	12.4	–7.3	6.7	14.3	7.6	8.6	7.3	–1.3	6.2	9	2.8	5.5	5	–0.5	6.9	10.8	3.9	47.4	74	26.6^e^
Received income	42	45.3	3.3	28.4	32.5	4.1	28.6	23.1	–5.5^e^	12.2	18.4	6.2^e^	3.9	4.2	0.3	4.1	4.8	0.7	11	8.7	–2.3	0.7	1.3	0.6	61.4	88.1	26.7^e^

a A&E: accident and emergency.

b T1: 2013.

c T2: 2023.

d % Difference

e *P*<.05.

f Not available.

### Sociodemographic and Clinical Correlates of Health Care Service Use in 2013 and 2023

Figure 1 presents the significant sociodemographic and clinical correlates of each type of health care service use in 2013 and 2023, whereas Multimedia Appendix 2 shows the complete set of sociodemographic and clinical factors for each health care service use in both years. We found that certain sociodemographic and clinical factors remained significantly associated with health care service use over time. For example, after adjusting for all covariates in the multivariable logistic regression analysis, those of Malay ethnicity (vs Chinese) were less likely to consult primary care doctors, while those who had HBP and diabetes were more likely to consult primary care doctors in both 2013 and 2023. We found that those diagnosed with dementia or heart problems and those retired (vs employed) were more likely to consult public hospital doctors and health workers in both 2013 and 2023. We also found that those diagnosed with dementia were more likely to use intermediate and long-term care in both 2013 and 2023. Furthermore, those of Malay and Indian ethnicity (vs Chinese) and with lower education (none, some, primary vs tertiary) were less likely to consult public and private dentists in both 2013 and 2023.

**Figure 1.**
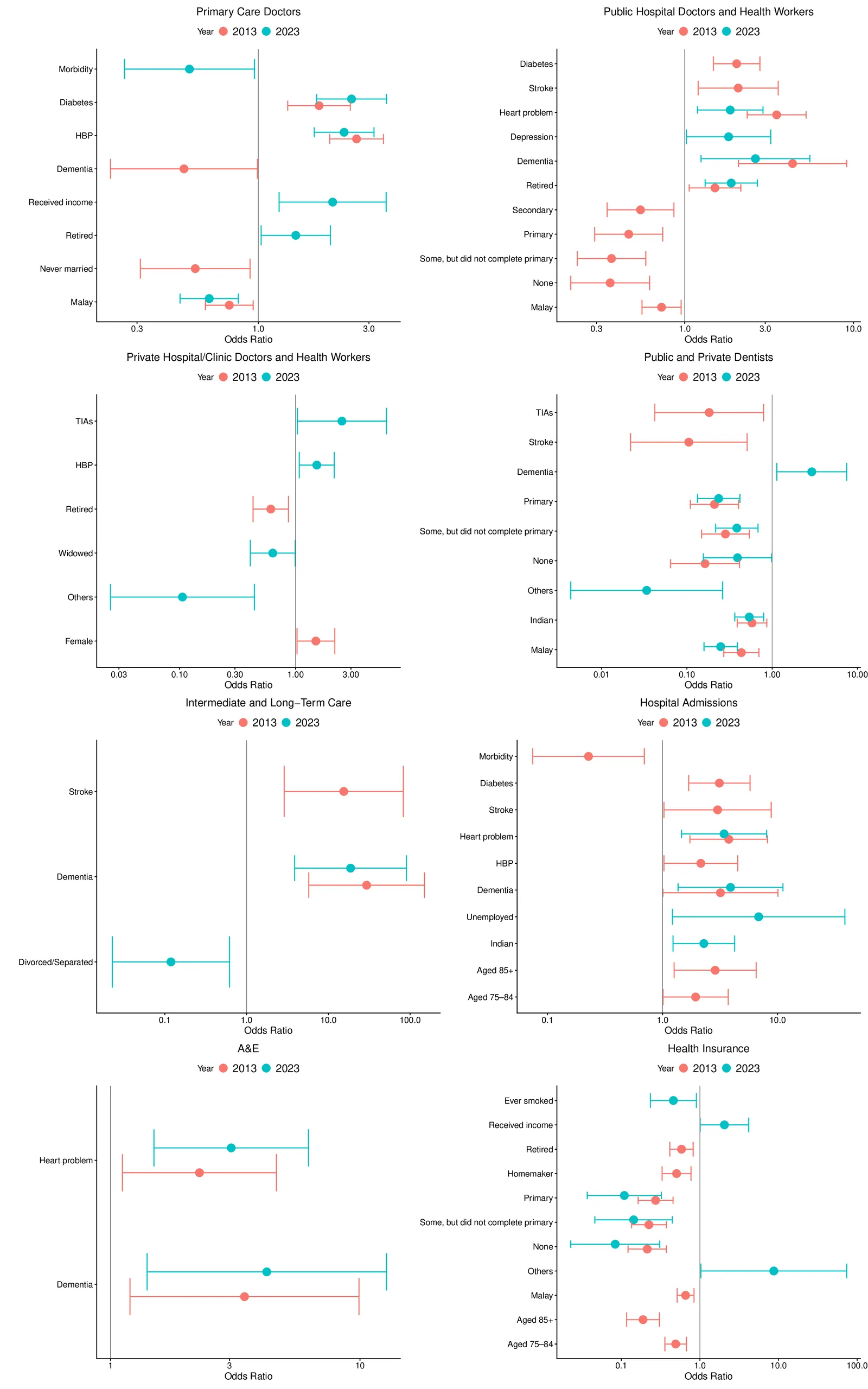
Significant sociodemographic and clinical correlates of each health care service use in 2013 and 2023. A&E: accident and emergency; HBP: high blood pressure; TIA: transient ischemic attack.

### The Network Structure of Health Care Service Use in 2013 and 2023

Figure 2 shows the pattern of the network structure of health care service use among older adults in 2013 and 2023, while Multimedia Appendix 3 shows the 95% bootstrapped CIs for all edge weights or connections among health care services. Each node in the network depicts a health care service use, and each edge represents a bidirectional partial relationship between services, after controlling for all other associations in the network. In 2023, there was a strong positive connection between the use of emergency department visits and hospital admissions, followed by strong connections between hospital admissions and respite care and nursing homes and between MediShield and ElderShield insurance. In 2013, a strong positive connection was observed between emergency department visits and hospital admissions, followed by strong positive connections between MediShield and ElderShield and between primary care doctor and medication use.

**Figure 2.**
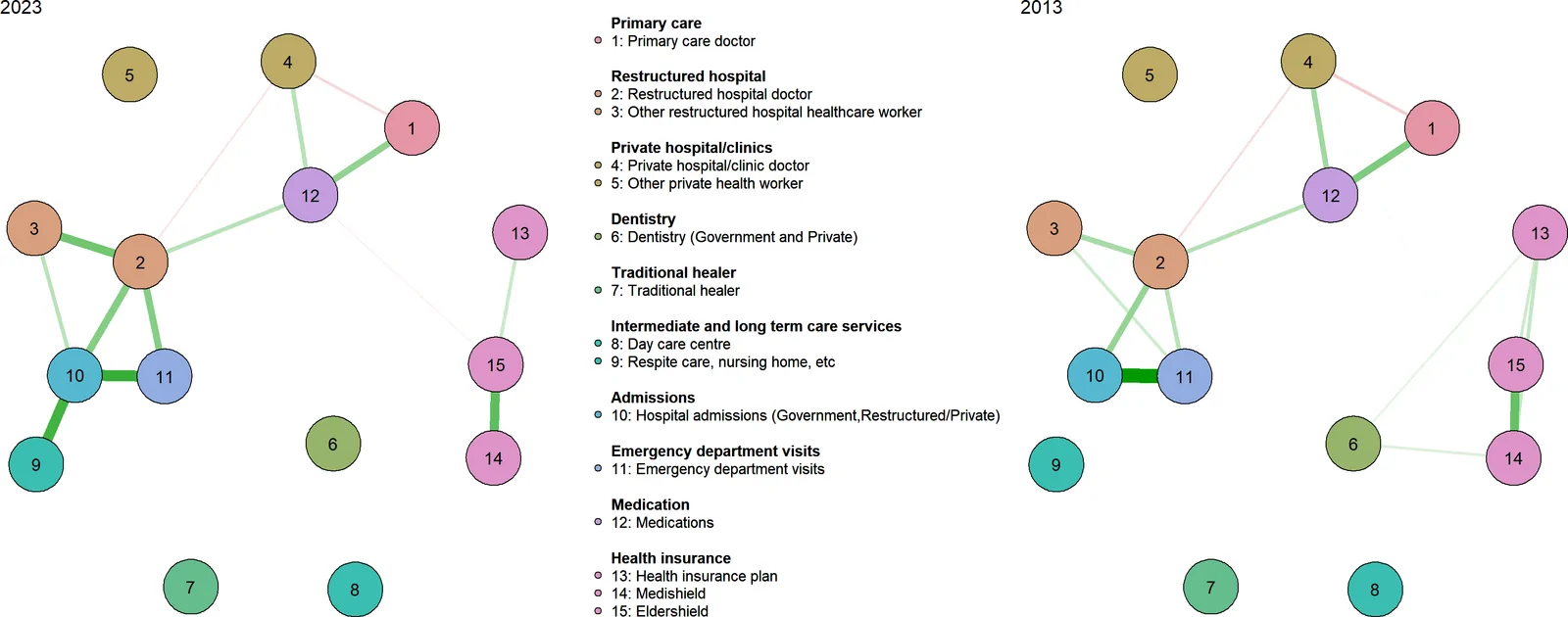
The pattern of the network structure of health care service use among older adults in 2013 and 2023.

## Discussion

### Principal Findings

In this study, we compared 2 nationwide cross-sectional surveys of older adults conducted 10 years apart and identified three main findings. First, the types of health care older adults used changed over the decade. Fewer people went to private hospitals or clinics, while more people used dental services and were covered by national health insurance and long-term care insurance. Second, the factors associated with health care use were broadly consistent across the 2 surveys; health problems such as dementia, HBP, and diabetes were the strongest correlates of service use. Meanwhile, certain groups such as Malays, Indians, and people with lower education continued to face gaps in dental and preventive care. Third, the network structure of health care use changed over the decade, showing stronger and more stable connections among services in 2023. Strong networks appeared between respite care, nursing homes, and hospital admissions, showing that these services were being used together more often in 2023.

Our results align closely with the Andersen Behavioral Model of Health Services Use [7], where need factors such as dementia, hypertension, and diabetes were the strongest and most consistent predictors of service use across both survey waves, while predisposing factors such as ethnicity and education explained persistent disparities in dental and preventive care use. The substantial expansion of enabling factors over the decade, particularly with MediShield Life and CareShield Life coverage, corresponds directly with the observed population-level shifts in insurance uptake and the reduction in private provider use.

The findings of this study have relevance beyond Singapore. Singapore’s experience offers valuable lessons, especially for other rapidly aging middle- and high-income settings, including South Korea, Taiwan, Japan, and parts of the Middle East, where demographic aging is accelerating but repeated, population-level data on health care usage patterns remain sparse. Four insights may have broad generalizability. First, mandatory enrollment in basic health insurance such as MediShield Life, combined with expanded voluntary uptake of long-term care insurance among older cohorts, can rapidly and substantially expand population-level coverage within a short policy time frame [19,20]. Second, expansion of health care subsidies and financial incentives can substantially shift use patterns across public and private sectors, highlighting how strongly cost drives health care decisions [19,20]. Third, network analysis provides a valuable and underused tool for detecting structural changes in care integration pathways at the population level. Finally, the persistent ethnic disparities in dental service use observed in this study are relevant to multiethnic societies globally, where minority populations continue to face access barriers that policy interventions have not fully overcome [21].

### Changes in the Proportion of Health Care Use

This study examined population-level changes in health care use among the older adult population in Singapore between 2013 and 2023. Over this 10-year period, primary care doctors and public hospital doctors remained the most commonly used health care providers among older adults in Singapore, underscoring their central role in the health care delivery system in Singapore. We found that the proportion of older adults who visited private hospitals or clinic doctors declined significantly in 2023 (a decrease of 4.6 percentage points; *P*=.02), suggesting a shift in health care–seeking behavior. This trend may reflect the impact of public health initiatives, including expanded subsidies and improved accessibility and capacity of primary care services and community hospitals nationwide. The increasing reliance on public health care resources among older adults aligns with national strategies such as HealthierSG [19] and the expansion of Community Health Assist Scheme (CHAS) subsidies. Such a shift has the potential to enhance equity in health care access, reduce out-of-pocket expenditures, and optimize resource use. Policymakers should continue to monitor these trends to ensure that public health care services remain adequately resourced and responsive to the evolving needs of Singapore’s aging population.

Notably, we observed a significant increase in the use of dental services, likely driven by government subsidies for private clinics through the introduction of CHAS dental benefits in 2012. Additionally, we observed a substantial increase in health insurance coverage among older adults following the implementation of national health insurance schemes, including MediShield Life [22]. MediShield Life was introduced on November 1, 2015, and covers all Singapore Citizens and Permanent Residents. The marked increase in long-term care insurance coverage in 2023 (27.5 percentage points) may reflect both continued ElderShield coverate among younger cohorts, and voluntary uptake of CarShiled Life along generous subsidies and incentives may have further increased long-term care insurance coverage.

Emergency department visits and hospitalizations were among the least frequently used direct medical services among older adults in Singapore. The proportion of hospitalizations (4.5% in 2013 and 4.4% in 2023) in our study was slightly higher than that in South Korea (2%) and lower than in Mexico and the United States (>5%) [23]. The lower rates of hospitalizations and emergency department visits among the older adult population may reflect the combined effects of system-level and population-level factors, including improvements in the quality of primary care as well as broader health determinants, population health improvements, expanded insurance coverage, and changes in care integration [19,24]. Previous studies have suggested that stronger primary care systems are generally associated with better population health outcomes, including lower rates of hospitalization and mortality [25,26]. These findings highlight the importance of continued investment in primary care to support healthy aging and reduce the burden on acute care services.

### Subgroup-Level Changes and Factors Associated With Health Care Use

In the multivariate analysis, we found that older adults with high blood pressure and diabetes were more likely to consult primary care doctors in both 2013 and 2023, suggesting a significant role of primary care in treating patients with these conditions. In Singapore, primary care institutions have adopted the Chronic Care Model (CCM) for care delivery [27]. Previous studies have reported that the CCM is an effective framework for improving the quality of diabetes and hypertension care in primary care settings [28]. Moreover, diabetes is one of the chronic conditions projected to increase health care expenditure and lead to greater productivity losses in Singapore [29] due to an uptrend in diabetes-related complications [30]. Hence, improving service delivery and increasing awareness through ongoing national campaigns such as the “War on Diabetes” [31] to promote active lifestyles, early screening and intervention, and better disease control should continue to combat diabetes and help the older adult population live healthier.

Our findings showed that those diagnosed with dementia and heart problems were more likely to consult public hospital doctors and health care workers in both 2013 and 2023. We also found that those diagnosed with dementia were more likely to visit intermediate and long-term care services in both 2013 and 2023. Dementia is a complex condition that requires a multidisciplinary team with specialized expertise and resources, including geriatric specialists from public hospitals. It has been suggested that patients with dementia are frequently admitted as emergency cases [32,33]. Similarly, heart problems are complex conditions that often require cardiologists and specialized services, such as cardiac rehabilitation programs that are available in public hospitals.

Malay older adults were consistently less likely to consult primary care doctors and public or private dentists in both 2013 and 2023. They were also less likely to use public hospital doctors and health workers and health insurance in 2013. This pattern suggests lower overall health care use among Malay older adults compared to their Chinese counterparts, potentially reflecting disparities in health literacy, life expectancy, and cultural differences in health beliefs and health-seeking behavior. For example, the National Population Health Survey 2024 [34] reported that the Malay ethnic group consistently exhibited the lowest rates of screening uptake for diabetes, hypertension, and high blood cholesterol compared to their Chinese and Indian counterparts [34]. Guo et al [35] similarly projected that the lowest lifetime healthcare expenditure among Malay older adults, despite a higher chronic disease burden, which attributed to shorter life expectancy. In contrast, Indian older adults were more likely than their Chinese counterparts to be admitted in 2023. This finding is consistent with Guo et al [35] projection that lifetime healthcare expenditure would be the highest among Indian older adults in Singapore, which may be attributed to the relatively higher prevalence of chronic medical conditions such as diabetes and hypertension in this group [36,37], as well as lifestyle factors.

Older adults with lower education showed a larger reduction in private hospital/clinic consultations and a greater increase in government and private dental consultations, as well as intermediate and long-term care. These patterns suggest a shift away from private providers toward subsidized public services, likely influenced by CHAS dental benefits and expanded subsidies under HealthierSG. The increased use of long-term care reflects the vulnerability of less educated groups, underscoring the importance of schemes like CHAS dental benefits and HealthierSG in reducing barriers to accessing care. Importantly, these initiatives are supported by a multipronged communication effort. For example, eligible seniors often receive direct mail and SMS invitations to enroll in HealthierSG and to understand their CHAS, PG, and MG card benefits. Information is also disseminated through official government websites (eg, Ministry of Health [MOH] and HealthierSG), as well as the HealthHub and Healthy 365 mobile apps, which provide digital health plans and activity tracking. At the same time, traditional methods remain crucial for reaching less tech-savvy seniors, complemented by dedicated helplines such as the MOH hotline and the CHAS hotline, which offer personalized assistance for enrollment and subsidy queries.

### Network Analyses

Our network analysis shows that there were strong connections among emergency department visits, hospital admissions, and seeing public hospital doctors in both 2013 and 2023. Interestingly, we only observed strong connections between respite care and nursing homes and hospital admissions in 2023. This finding is consistent with a previous study, which indicated that higher hospital admissions and transfers to hospital for acute care were common among nursing home residents [38]. This suggests a significant shift in service use, which warrants further investigation. Bootstrapped CI findings showed that there were more significant and stable network connections between health care services in 2023 than in 2013. This finding could be related to the broadened health care coverage and expansion of care integration between multidisciplinary teams from primary care, public hospitals, and intermediate and long-term care institutions for the older adult population in Singapore. However, further studies using network analysis from a qualitative perspective might be needed to better understand this relationship [39].

### Limitations and Strengths

The present study has some limitations that must be considered while interpreting the findings. First, data on direct health care use were self-report, which may be influenced by recall and memory biases. Second, the edge weights in the network analysis, which represent the strength of co-utilization between health care services, may be influenced by small sample sizes. Health care services with low usage (eg, day care, respite care, or intermediate and long-term care) were reported by relatively few respondents, which could affect the stability and interpretability of their associated edges. While all edges reflect observed co-occurrence patterns, those derived from smaller subsamples should be interpreted with caution. To address this, we applied bootstrapping to enhance the robustness of our estimates. Notwithstanding the limitations, the present study has notable strengths: the data comprise 2 large, nationally representative, multiethnic samples, and a comprehensive assessment of service use was conducted using standardized instruments for comparability with other population-based studies.

### Conclusions

Our study findings address important gaps in the literature on health care use in aging populations. To our knowledge, this is one of the few studies to provide repeated cross-sectional, population-level estimates of direct health care service use a decade apart in a multiethnic Asian older adult population and the first to apply network analysis methods to understand the links between different types of health care services used by older adults in Singapore. These findings contribute to 3 bodies of evidence: health care system responsiveness to population aging, equity research on persistent and emergent disparities in service access, and methodological work on network analysis approaches to understanding care integration. Future research should examine whether observed trends are sustained and whether care integration initiatives translate into improved health outcomes for older adults.

## Supplementary material

10.2196/80157 Multimedia Appendix 1 Sociodemographic distribution of the older adults in 2013 and 2023.

10.2196/80157 Multimedia Appendix 2 Sociodemographic and clinical correlates of health care service use in 2013 and 2023.

10.2196/80157 Multimedia Appendix 3 95% bootstrapped CI results for all edge weights or connections among health care service use.

## Abbreviations

CCM: Chronic Care Model
CHAS: Community Health Assist Scheme
CSRI: Client Service Receipt Inventory
DSRB: Domain Specific Review Board
GP: general practitioner
HBP: high blood pressure
MCA: Ministerial Committee on Aging
MCI: mild cognitive impairment
MG: Merdeka Generation
MOH: Ministry of Health
NHG: National Health Care Group
PG: Pioneer Generation
TIA: transient ischemic attack
WiSE: Wellbeing of the Singapore Elderly

## References

[1] United Nations Department of Economic and Social Affairs, Population Division (2022). World population prospects 2022: summary of results. https://www.un.org/development/desa/pd/sites/www.un.org.development.desa.pd/files/wpp2022_summary_of_results.pdf.

[2] Chen P, Cai H, Bai W (2023). Global prevalence of mild cognitive impairment among older adults living in nursing homes: a meta-analysis and systematic review of epidemiological surveys. Transl Psychiatry.

[3] Bai W, Chen P, Cai H (2022). Worldwide prevalence of mild cognitive impairment among community dwellers aged 50 years and older: a meta-analysis and systematic review of epidemiology studies. Age Ageing.

[4] GBD 2019 Dementia Forecasting Collaborators (2022). Estimation of the global prevalence of dementia in 2019 and forecasted prevalence in 2050: an analysis for the Global Burden of Disease Study 2019. Lancet Public Health.

[5] Kadambi S, Abdallah M, Loh KP (2020). Multimorbidity, function, and cognition in aging. Clin Geriatr Med.

[6] Bierman AS, Wang J, O’Malley PG, Moss DK (2021). Transforming care for people with multiple chronic conditions: Agency for Healthcare Research and Quality’s research agenda. Health Serv Res.

[7] Andersen RM (1995). Revisiting the behavioral model and access to medical care: does it matter?. J Health Soc Behav.

[8] Yin Z, Stratton L, Song Q (2024). Perceptions and utilization of online peer support among informal dementia caregivers: survey study. JMIR Aging.

[9] (2023). 2023 action plan for successful ageing. https://isomer-user-content.by.gov.sg/3/b1fd5713-8ff9-46d5-9911-0f233f2a8b31/refreshed-action-plan-for-successful-ageing-2023.pdf.

[10] Longevity. National Population and Talent Division.

[11] Schleich AM (2024). Perspectives of Two Island Nations: Singapore – New Zealand.

[12] Lim MK (2005). Transforming Singapore health care: public-private partnership. Ann Acad Med Singap.

[13] Foo CD, Woon TH, Chia HX (2025). Healthier SG: a gateway for evolving public-private-population partnerships in population health. Lancet Reg Health West Pac.

[14] Subramaniam M, Chong SA, Vaingankar JA (2015). Prevalence of dementia in people aged 60 years and above: results from the WiSE study. J Alzheimers Dis.

[15] Subramaniam M, Abdin E, Asharani PV (2025). Prevalence of dementia in Singapore: changes across a decade. Alzheimers Dement.

[16] Beecham J, Knapp M, Thornicroft G, Brewin CR, Wing J (1992). Measuring Mental Health Needs.

[17] van Borkulo CD, Borsboom D, Epskamp S (2014). A new method for constructing networks from binary data. Sci Rep.

[18] Epskamp S, Cramer AOJ, Waldorp LJ, Schmittmann VD, Borsboom D (2012). qgraph: network visualizations of relationships in psychometric data. J Stat Soft.

[19] Foo CD, Chia HX, Teo KW (2023). Healthier SG: Singapore’s multi-year strategy to transform primary healthcare. Lancet Reg Health West Pac.

[20] (2022). White paper on healthier SG. https://file.go.gov.sg/healthiersg-whitepaper-pdf.pdf.

[21] Chen AM (2025). Practical barriers to health equity: challenges, opportunities, and solutions. Adv Public Health.

[22] Earn LC (2020). International profiles of health care systems, 2020. https://www.commonwealthfund.org/sites/default/files/2020-12/International_Profiles_of_Health_Care_Systems_Dec2020.pdf.

[23] Macinko J, Cristina Drumond Andrade F, Bof de Andrade F, Lima-Costa MF (2020). Universal health coverage: are older adults being left behind? Evidence from aging cohorts in twenty-three countries. Health Aff (Millwood).

[24] (2025). Expanding healthcare capacity and transforming the healthcare workforce. Ministry of Health, Singapore.

[25] Shi L (2012). The impact of primary care: a focused review. Scientifica (Cairo).

[26] Niti M, Ng TP (2003). Avoidable hospitalisation rates in Singapore, 1991-1998: assessing trends and inequities of quality in primary care. J Epidemiol Community Health.

[27] Zheng LF, Koh YLE, Sankari U, Tan NC (2019). Asthma care based on Chronic Care Model in an aging Asian community. NPJ Prim Care Respir Med.

[28] Goh LH, Siah CJR, Tam WWS, Tai ES, Young DYL (2022). Effectiveness of the chronic care model for adults with type 2 diabetes in primary care: a systematic review and meta-analysis. Syst Rev.

[29] Png ME, Yoong J, Phan TP, Wee HL (2016). Current and future economic burden of diabetes among working-age adults in Asia: conservative estimates for Singapore from 2010-2050. BMC Public Health.

[30] Tan JK, Salim NNM, Lim GH, Chia SY, Thumboo J, Bee YM (2022). Trends in diabetes-related complications in Singapore, 2013-2020: a registry-based study. PLoS One.

[31] (2016). Speech by minister for health, Mr Gan Kim Yong, at the MOH Committee of Supply debate 2016. Ministry of Health, Singapore.

[32] Natalwala A, Potluri R, Uppal H, Heun R (2008). Reasons for hospital admissions in dementia patients in Birmingham, UK, during 2002-2007. Dement Geriatr Cogn Disord.

[33] Gerlach LB, Martindale J, Bynum JPW, Davis MA (2023). Characteristics of emergency department visits among older adults with dementia. JAMA Neurol.

[34] (2025). National population health survey 2024 (household interview and health examination). https://isomer-user-content.by.gov.sg/3/38583351-2fdc-4a01-8bf7-88d24889af78/NPHS%202024%20Survey%20Report.pdf.

[35] Guo X, Tysinger B, Wee HL (2025). Disease burden, lifetime healthcare cost and long-term intervention impact projections among older adults in Singapore. Nat Aging.

[36] Seow LSE, Subramaniam M, Abdin E, Vaingankar JA, Chong SA (2015). Hypertension and its associated risks among Singapore elderly residential population. Journal of Clinical Gerontology and Geriatrics.

[37] Yeo KK, Tai BC, Heng D (2006). Ethnicity modifies the association between diabetes mellitus and ischaemic heart disease in Chinese, Malays and Asian Indians living in Singapore. Diabetologia.

[38] Goh HS, Tan S, Khaw AK, Ow CC, Devi MK (2018). Hospital admissions among nursing home residents in Singapore: a retrospective study. Singapore Nurs J.

[39] Vos J, Gerling K, Linehan C, Siriwardena AN, Windle K (2018). Understanding care navigation by older adults with multimorbidity: mixed-methods study using social network and framework analyses. JMIR Aging.

