# Supplementary material for: Changes in Patterns of Health Care Use Among Older Adults in Singapore Across a Decade: Repeated Nationwide Cross-Sectional Survey
Source: JMIR Aging. 2026 Nov 5;9:e80157. doi: 10.2196/80157 (PMC13738006; doi:10.2196/80157)
Supplement: Supplementary file 1 [file aging-v9-e80157-s001.docx]

Supplementary Table 1: Sociodemographic distribution of the older adults in 2013 and 2023

|  |  | 2013 | | 2023 | | p value |
| --- | --- | --- | --- | --- | --- | --- |
|  |  | n | % | n | % |  |
| Age group | 60-74 | 1494 | 75.0 | 1830 | 75.0 | 0.580 |
|  | 75-84 | 669 | 19.5 | 172 | 18.7 |  |
|  | 85+ | 402 | 5.5 | 340 | 6.3 |  |
| Gender | Men | 1117 | 44.1 | 908 | 47.1 | 0.174 |
|  | Women | 1448 | 55.9 | 1102 | 52.9 |  |
| Ethnicity | Chinese | 1012 | 83.3 | 678 | 81.6 | 0.632 |
|  | Malay | 745 | 9.3 | 699 | 10.4 |  |
|  | Indian | 772 | 6.0 | 616 | 6.4 |  |
|  | Others | 36 | 1.4 | 17 | 1.5 |  |
| Education | None | 511 | 16.5 | 217 | 7.2 | **0.000** |
|  | Some, but did not complete primary | 620 | 23.9 | 391 | 20.6 |  |
|  | Primary | 640 | 24.8 | 568 | 26.5 |  |
|  | Secondary | 517 | 22.4 | 545 | 28.2 |  |
|  | Tertiary | 262 | 12.4 | 282 | 17.6 |  |
| Marital | Never married | 136 | 8.0 | 114 | 9.1 | 0.158 |
|  | Married | 1484 | 64.0 | 1168 | 66.2 |  |
|  | Widowed | 836 | 22.5 | 617 | 18.7 |  |
|  | Divorced/Separated | 107 | 5.5 | 107 | 6.0 |  |
| Employment | Employed | 688 | 33.9 | 633 | 42.2 | **0.000** |
|  | Unemployed | 32 | 1.5 | 35 | 1.3 |  |
|  | Homemaker | 808 | 26.3 | 515 | 16.8 |  |
|  | Retired | 1006 | 38.3 | 821 | 39.7 |  |
| Received other income | No | 372 | 10.7 | 135 | 6.3 | **0.000** |
|  | Yes | 2188 | 89.3 | 1864 | 93.7 |  |
