# Supplementary material for: Changes in Patterns of Health Care Use Among Older Adults in Singapore Across a Decade: Repeated Nationwide Cross-Sectional Survey
Source: JMIR Aging. 2026 Nov 5;9:e80157. doi: 10.2196/80157 (PMC13738006; doi:10.2196/80157)
Supplement: Supplementary file 2 [file aging-v9-e80157-s002.docx]

Supplementary Table 2. Sociodemographic and clinical correlates of healthcare service use in 2013 and 2023

|  | Primary care doctors | | | | Public hospital doctor and health workers | | | | Private hospital/clinic doctors and health workers | | | | Public and private dentists | | | |
| --- | --- | --- | --- | --- | --- | --- | --- | --- | --- | --- | --- | --- | --- | --- | --- | --- |
|  | 2013 | | 2023 | | 2013 | | 2023 | | 2013 | | 2023 | | 2013 | | 2023 | |
|  | OR | p | OR | p | OR | p | OR | p | OR | p | OR | p | OR | p | OR | p |
| 60-74 | 1.00 |  | 1.00 |  | 1.00 |  | 1.00 |  | 1.00 |  | 1.00 |  | 1.00 |  | 1.00 |  |
| 75-84 | 1.17 | 0.319 | 0.86 | 0.452 | 1.23 | 0.227 | 1.24 | 0.295 | 1.07 | 0.672 | 1.30 | 0.242 | 0.87 | 0.608 | 0.64 | 0.095 |
| 85+ | 1.04 | 0.865 | 0.75 | 0.316 | 1.12 | 0.628 | 1.28 | 0.392 | 1.14 | 0.556 | 1.59 | 0.160 | 0.49 | 0.121 | 0.49 | 0.096 |
| Female | 0.98 | 0.911 | 0.99 | 0.942 | 1.40 | 0.080 | 1.06 | 0.773 | 1.50 | **0.035** | 1.37 | 0.151 | 1.03 | 0.917 | 0.94 | 0.781 |
| Chinese | 1.00 |  | 1.00 |  | 1.00 |  | 1.00 |  | 1.00 |  | 1.00 |  | 1.00 |  | 1.00 |  |
| Malay | 0.75 | **0.017** | 0.61 | **0.001** | 0.72 | **0.017** | 0.85 | 0.289 | 0.92 | 0.510 | 0.87 | 0.390 | 0.44 | **0.001** | 0.25 | **0.000** |
| Indian | 1.11 | 0.410 | 1.14 | 0.369 | 1.01 | 0.954 | 1.16 | 0.311 | 0.83 | 0.165 | 1.17 | 0.341 | 0.58 | **0.008** | 0.54 | **0.002** |
| Others | 0.93 | 0.841 | 0.83 | 0.863 | 1.13 | 0.755 | 2.21 | 0.640 | 1.61 | 0.228 | 0.11 | **0.002** | 1.33 | 0.594 | 0.03 | **0.001** |
| Never married | 0.53 | **0.024** | 1.03 | 0.920 | 0.70 | 0.258 | 0.80 | 0.444 | 0.72 | 0.244 | 1.02 | 0.948 | 1.32 | 0.447 | 0.69 | 0.329 |
| Married | 1.00 |  | 1.00 |  | 1.00 |  | 1.00 |  | 1.00 |  | 1.00 |  | 1.00 |  | 1.00 |  |
| Separated | 0.86 | 0.389 | 1.08 | 0.726 | 0.81 | 0.277 | 0.86 | 0.500 | 0.78 | 0.182 | 0.64 | **0.047** | 0.85 | 0.612 | 1.03 | 0.923 |
| Divorced/Widowed | 0.92 | 0.772 | 1.01 | 0.973 | 1.23 | 0.491 | 0.90 | 0.741 | 0.54 | 0.088 | 0.52 | 0.099 | 0.52 | 0.259 | 0.87 | 0.739 |
| None | 1.35 | 0.237 | 0.83 | 0.583 | 0.36 | **0.000** | 0.61 | 0.166 | 0.65 | 0.109 | 1.15 | 0.693 | 0.16 | **0.000** | 0.39 | **0.047** |
| Some, but did not  complete primary | 1.43 | 0.120 | 0.94 | 0.791 | 0.37 | **0.000** | 0.66 | 0.097 | 0.82 | 0.416 | 0.72 | 0.249 | 0.28 | **0.000** | 0.39 | **0.001** |
| Primary | 1.30 | 0.254 | 1.23 | 0.361 | 0.47 | **0.001** | 0.92 | 0.711 | 0.69 | 0.112 | 0.89 | 0.634 | 0.21 | **0.000** | 0.24 | **0.000** |
| Secondary | 1.31 | 0.233 | 1.31 | 0.217 | 0.55 | **0.009** | 0.84 | 0.429 | 0.86 | 0.511 | 0.76 | 0.267 | 0.79 | 0.379 | 0.60 | 0.050 |
| Tertiary | 1.00 |  | 1.00 |  | 1.00 |  | 1.00 |  | 1.00 |  | 1.00 |  | 1.00 |  | 1.00 |  |
| Employed | 1.00 |  | 1.00 |  | 1.00 |  | 1.00 |  | 1.00 |  | 1.00 |  | 1.00 |  | 1.00 |  |
| Unemployed | 2.99 | 0.052 | 2.17 | 0.140 | 2.14 | 0.200 | 2.19 | 0.174 | 0.95 | 0.936 | 0.43 | 0.118 | 1.69 | 0.520 | 1.26 | 0.752 |
| Homemaker | 0.82 | 0.316 | 1.38 | 0.169 | 1.21 | 0.399 | 1.23 | 0.400 | 0.94 | 0.773 | 1.16 | 0.574 | 0.58 | 0.114 | 1.56 | 0.134 |
| Retired | 1.23 | 0.205 | 1.45 | **0.034** | 1.51 | **0.022** | 1.89 | **0.000** | 0.61 | **0.006** | 0.92 | 0.665 | 0.69 | 0.131 | 0.84 | 0.443 |
| Received income | 0.94 | 0.759 | 2.09 | **0.007** | 1.02 | 0.910 | 1.86 | 0.058 | 1.51 | 0.092 | 2.18 | 0.062 | 1.37 | 0.481 | 1.42 | 0.378 |
| Dementia | 0.48 | **0.047** | 1.20 | 0.623 | 4.37 | **0.000** | 2.62 | **0.011** | 0.61 | 0.193 | 0.72 | 0.491 | 0.68 | 0.553 | 2.92 | **0.026** |
| Depression | 0.77 | 0.273 | 1.57 | 0.103 | 1.30 | 0.300 | 1.82 | **0.041** | 1.41 | 0.165 | 1.26 | 0.453 | 1.68 | 0.140 | 0.92 | 0.795 |
| HBP | 2.65 | **0.000** | 2.34 | **0.000** | 1.17 | 0.289 | 1.01 | 0.936 | 1.27 | 0.112 | 1.52 | **0.017** | 1.25 | 0.314 | 1.18 | 0.422 |
| Heart problem | 0.88 | 0.542 | 1.45 | 0.095 | 3.52 | **0.000** | 1.86 | **0.007** | 1.03 | 0.899 | 0.87 | 0.606 | 1.27 | 0.472 | 1.68 | 0.066 |
| Stroke | 1.04 | 0.883 | 1.19 | 0.593 | 2.06 | **0.009** | 1.12 | 0.745 | 0.87 | 0.683 | 0.49 | 0.082 | 0.11 | **0.005** | 0.56 | 0.235 |
| TIAs | 0.85 | 0.676 | 0.97 | 0.947 | 0.85 | 0.734 | 1.29 | 0.582 | 0.49 | 0.209 | 2.51 | **0.041** | 0.18 | **0.023** | 0.52 | 0.350 |
| Diabetes | 1.83 | **0.000** | 2.52 | **0.000** | 2.03 | **0.000** | 0.76 | 0.143 | 1.11 | 0.545 | 0.93 | 0.710 | 1.14 | 0.626 | 1.32 | 0.234 |
| Morbidity | 1.08 | 0.804 | 0.50 | **0.038** | 0.67 | 0.198 | 1.45 | 0.268 | 0.77 | 0.448 | 0.77 | 0.491 | 1.86 | 0.205 | 0.91 | 0.826 |
| Ever smoked | 0.73 | 0.053 | 1.40 | 0.092 | 0.89 | 0.500 | 0.98 | 0.912 | 1.21 | 0.279 | 1.18 | 0.477 | 0.65 | 0.116 | 0.87 | 0.616 |

Continue. Supplementary Table 3. Sociodemographic and clinical correlates of healthcare service use in 2013 and 2023

|  | Intermediate and long-term care | | | | Hospital admissions | | | | A&E | | | | Health insurance | |  |  |
| --- | --- | --- | --- | --- | --- | --- | --- | --- | --- | --- | --- | --- | --- | --- | --- | --- |
|  | 2013 | | 2023 | | 2013 | | 2023 | | 2013 | | 2023 | | 2013 | | 2023 | |
|  | OR | p | OR | p | OR | p | OR | p | OR | p | OR | p | OR | p | OR | p |
| 60-74 | 1.00 |  | 1.00 |  | 1.00 |  | 1.00 |  | 1.00 |  | 1.00 |  | 1.00 |  | 1.00 |  |
| 75-84 | 0.34 | 0.151 | 3.55 | 0.119 | 1.94 | **0.047** | 1.28 | 0.533 | 0.81 | 0.560 | 1.91 | 0.130 | 0.49 | **0.000** | 0.91 | 0.753 |
| 85+ | 1.56 | 0.487 | 2.66 | 0.433 | 2.87 | **0.012** | 2.10 | 0.202 | 1.54 | 0.323 | 2.32 | 0.137 | 0.19 | **0.000** | 1.44 | 0.405 |
| Female | 0.48 | 0.453 | 3.46 | 0.105 | 0.72 | 0.449 | 0.58 | 0.171 | 1.21 | 0.658 | 1.25 | 0.591 | 0.81 | 0.282 | 0.56 | 0.069 |
| Chinese | 1.00 |  | 1.00 |  | 1.00 |  | 1.00 |  | 1.00 |  | 1.00 |  | 1.00 |  | 1.00 |  |
| Malay | 0.77 | 0.694 | 1.39 | 0.549 | 1.03 | 0.905 | 1.40 | 0.282 | 0.82 | 0.496 | 0.91 | 0.774 | 0.66 | **0.001** | 1.07 | 0.762 |
| Indian | 0.76 | 0.670 | 1.94 | 0.203 | 0.98 | 0.946 | 2.29 | **0.009** | 1.18 | 0.521 | 1.66 | 0.104 | 0.95 | 0.703 | 1.15 | 0.542 |
| Others | 1.00 |  | 0.61 | 0.565 | 0.96 | 0.970 | 1.00 |  | 1.44 | 0.592 | 1.00 |  | 1.26 | 0.555 | 8.70 | **0.048** |
| Never married | 1.00 |  | 2.42 | 0.328 | 0.70 | 0.564 | 0.70 | 0.659 | 0.50 | 0.416 | 0.99 | 0.991 | 0.79 | 0.375 | 0.68 | 0.346 |
| Married | 1.00 |  | 1.00 |  | 1.00 |  | 1.00 |  | 1.00 |  | 1.00 |  | 1.00 |  | 1.00 |  |
| Separated | 1.63 | 0.475 | 0.36 | 0.197 | 0.52 | 0.107 | 0.42 | 0.054 | 0.86 | 0.691 | 1.04 | 0.928 | 0.97 | 0.846 | 0.75 | 0.312 |
| Divorced/Widowed | 0.48 | 0.584 | 0.12 | **0.011** | 0.81 | 0.792 | 0.38 | 0.278 | 1.22 | 0.779 | 0.51 | 0.374 | 0.98 | 0.943 | 1.15 | 0.779 |
| None | 3.49 | 0.326 | 1.43 | 0.672 | 1.00 | 0.998 | 2.07 | 0.261 | 1.86 | 0.257 | 0.52 | 0.331 | 0.21 | **0.000** | 0.08 | **0.000** |
| Some, but did not  complete primary | 1.77 | 0.623 | 1.00 | 0.996 | 0.60 | 0.322 | 1.38 | 0.587 | 1.02 | 0.972 | 0.76 | 0.602 | 0.22 | **0.000** | 0.14 | **0.001** |
| Primary | 7.01 | 0.094 | 0.34 | 0.184 | 1.28 | 0.623 | 1.08 | 0.876 | 0.95 | 0.924 | 0.71 | 0.423 | 0.27 | **0.000** | 0.11 | **0.000** |
| Secondary | 2.96 | 0.384 | 1.67 | 0.411 | 1.36 | 0.556 | 0.93 | 0.889 | 1.18 | 0.776 | 0.68 | 0.399 | 0.76 | 0.317 | 0.44 | 0.141 |
| Tertiary | 1.00 |  | 1.00 |  | 1.00 |  | 1.00 |  | 1.00 |  | 1.00 |  | 1.00 |  | 1.00 |  |
| Employed | 1.00 |  | 1.00 |  | 1.00 |  | 1.00 |  | 1.00 |  | 1.00 |  | 1.00 |  | 1.00 |  |
| Unemployed | 1.00 |  | 1.00 |  | 1.00 |  | 6.85 | **0.029** | 1.00 |  | 4.28 | 0.161 | 1.04 | 0.942 | 0.89 | 0.842 |
| Homemaker | 0.82 | 0.786 | 0.99 | 0.994 | 1.39 | 0.501 | 2.09 | 0.223 | 1.27 | 0.613 | 0.45 | 0.140 | 0.50 | **0.002** | 0.56 | 0.106 |
| Retired | 1.00 |  | 1.00 |  | 1.95 | 0.134 | 1.48 | 0.337 | 1.37 | 0.430 | 1.02 | 0.966 | 0.58 | **0.002** | 0.75 | 0.285 |
| Received income | 0.40 | 0.151 | 0.32 | 0.167 | 0.65 | 0.228 | 0.93 | 0.889 | 1.04 | 0.938 | 0.78 | 0.649 | 1.07 | 0.753 | 2.05 | **0.048** |
| Dementia | 30.12 | **0.000** | 18.69 | **0.000** | 3.18 | **0.049** | 3.90 | **0.011** | 3.44 | **0.022** | 4.23 | **0.011** | 1.01 | 0.974 | 1.79 | 0.221 |
| Depression | 1.02 | 0.980 | 0.47 | 0.378 | 2.06 | 0.163 | 0.80 | 0.713 | 1.34 | 0.567 | 1.47 | 0.494 | 1.10 | 0.692 | 1.27 | 0.627 |
| HBP | 1.36 | 0.646 | 2.11 | 0.216 | 2.15 | **0.042** | 1.47 | 0.297 | 1.36 | 0.378 | 1.61 | 0.216 | 1.14 | 0.344 | 0.67 | 0.076 |
| Heart problem | 0.59 | 0.546 | 0.58 | 0.434 | 3.76 | **0.001** | 3.43 | **0.005** | 2.27 | **0.024** | 3.05 | **0.002** | 0.99 | 0.955 | 0.79 | 0.491 |
| Stroke | 15.41 | **0.001** | 4.52 | 0.057 | 3.01 | **0.044** | 1.41 | 0.607 | 1.80 | 0.228 | 1.40 | 0.545 | 0.72 | 0.259 | 0.57 | 0.216 |
| TIAs | 0.15 | 0.184 | 0.23 | 0.197 | 3.48 | 0.106 | 1.74 | 0.413 | 0.82 | 0.731 | 2.92 | 0.095 | 0.72 | 0.431 | 0.44 | 0.126 |
| Diabetes | 0.95 | 0.952 | 0.63 | 0.596 | 3.12 | **0.000** | 0.64 | 0.294 | 1.47 | 0.270 | 0.53 | 0.130 | 0.76 | 0.091 | 1.06 | 0.810 |
| Morbidity | 0.50 | 0.602 | 4.27 | 0.142 | 0.23 | **0.009** | 1.35 | 0.597 | 0.76 | 0.608 | 1.11 | 0.863 | 0.99 | 0.984 | 1.65 | 0.317 |
| Ever smoked | 3.94 | 0.072 | 1.42 | 0.681 | 1.03 | 0.942 | 0.90 | 0.799 | 1.56 | 0.146 | 2.20 | 0.071 | 0.77 | 0.132 | 0.46 | **0.024** |
